# Age and Sex Ratios in a High-Density Wild Red-Legged Partridge Population

**DOI:** 10.1371/journal.pone.0159765

**Published:** 2016-08-10

**Authors:** Jesús Nadal, Carolina Ponz, Antoni Margalida

**Affiliations:** 1 Department of Animal Science, Division of Wildlife, Faculty of Life Sciences and Engineering, University of Lleida, Lleida, Spain; 2 Division of Conservation Biology, Institute of Ecology and Evolution, University of Bern, Bern, Switzerland; University of Minnesota, UNITED STATES

## Abstract

The dynamics of a wild red-legged partridge population were examined over a 14-year period in Spain to identify patterns in age and sex ratios in relation to weather parameters, and to assess the importance of these parameters in population dynamics and management. The results gave age ratios of 1.07 (but 2.13 in July counts), juvenile sex ratios of 1.01 and adult sex ratios of 1.47. Overall, 12% more females were hatched and female juvenile mortality was 7.3% higher than in males. Sex differential mortality explains the 19.2% deficit in adult females, which are more heavily predated than males during the breeding period. Accordingly, age ratios are dependent on sex ratios and both are density dependent. Over time, ratios and density changes appear to be influenced by weather and management. When the habitat is well conserved, partridge population dynamics can be explained by a causal chain: weather operates on net primary production, thereby affecting partridge reproduction and predation and, as a result, age and sex ratios in the October population. A reduction in the impact of predation (i.e. the effects of ground predators on eggs, chicks and breeding females) is the key factor to improve the conservation of partridge populations and associated biological processes.

## Introduction

Age and sex ratios change as annual partridge production and predation rates vary [1–3] and hence large age ratios indicate good annual partridge recruitment. Juvenile sex ratios are generally 1:1; however, adult sex ratios in birds are generally biased towards males but in mammals are frequently skewed towards females [4–6]. Different genetic, behavioural and ecological theories have been formulated to explain biased sex ratios during life stages [5–7], and characteristics ranging from genetic sex determination to parental condition, behaviour, environment and weather have all been postulated as the main factors capable of modulating juvenile sex ratios [8–12].

Age and sex ratios in partridge populations are still very poorly known [13–15]. The frequency and causes of variations in these ratios and the responses in population dynamics are all poorly understood and so the overall extent to which subtle changes in age and sex ratios can modify partridge densities is also unknown [13,16,17]. Understanding variations in age and sex ratios is extremely challenging because ratios vary seasonally, annually, spatially and between age and sex classes, and require clear definitions of what constitutes a sample: for instance, samples from smaller or larger spatial areas, or over shorter or longer time intervals, are interpretable in different ways [18–20].

The development of a simple and robust method for estimating age and sex ratios in the field, as well as for interpreting the results obtained, could improve wildlife management and conservation. Habitat quality (resource availability), density (competition), sexual size dimorphism (sex specific mortality), mating system (sexual selection, parental condition and capacity), synchrony (distribution of temporal opportunity) and predation (population regulation) affect offspring condition and production (chick numbers, quality and sex ratio) [4,6,21]. A better understanding of age and sex ratios in wild game bird populations could greatly improve our understanding of population processes and contribute to their management and conservation [22–24]

The increase in anthropogenic activities is having an impact on natural ecosystems and degrading biological processes [25,26]. Consequently, wildlife management has had to urgently establish conservation priorities as the degradation of ecosystems derives towards a loss of natural resources. In recent years, wild partridge populations have been replaced by farmed birds and game workers (managers, keepers, agents and businesses) have shifted their activities towards artificial production.

In red-legged partridges (*Alectoris rufa*), rapid habitat degradation has led to increased exposure of nests and coveys to predators and detrimental anthropogenic activities (i.e. agricultural machinery). This provokes greater mortality in young and female birds and thus consistently adult-skewed and male-skewed age and sex ratios in these populations [27]. Hence, in areas of better quality habitat, we can expect juvenile survival and age ratios to be higher, and also that male and female survival rates will be similar and sex ratios balanced [3,28,29]. In this sense, net primary production (NPP) depends on meteorological conditions but is also associated with the availability of food resources and predation risks. Both factors alter age and sex ratios and as consequence, their dynamics reflect a population responses to changes in annual habitat quality [3,30,31].

Annual, local and global age and sex ratios in declining red-legged partridge populations have been reported in northern Spain [14,27,32]. By contrast, age and sex ratios in stable or expanding populations are still very poorly studied and long time datasets are costly and difficult to obtain. Monitoring game bird populations requires robust methods for annual assessments of fluctuations in populations and in the resulting population dynamics [31,33,34]. Here, we take advantage of data from a long-term study (14 years) of a wild red-legged partridge population in La Mancha (Spain) to (1) describe current age and sex ratios in a high density population; (2) identify patterns of age and sex ratios and their causes, correlates and variations in order to respond the following questions: (a) Are age and sex ratio mutually dependent? (b) Are age and sex ratio density dependent? (c) Are age and sex ratio of year n+1 dependent on age and sex ratios of year n?; (3) assess the importance of known abundance, age and sex ratios in population monitoring, management and conservation to evaluate population health, stage and viability. (4) use and propose various statistical techniques for correctly interpreting annual age and sex ratios in high density populations; and (5) examine the effect of weather on age and sex ratios.

## Materials and Methods

### Ethical statement

The study was conducted in full compliance with Spanish laws and regulation, including the licence of “Las Ensanchas” to sampling shoot partridges. The protocol was approved by the Committee on the Ethics of Animal Experiments of the University of Lleida (Ref.1998-2012/05).

### Study area

The study was carried out in Las Ensanchas, a small-game hunting estate in the basin of the river Jabalón in Ciudad Real province (central Spain; 38°39’ N, 3°13’ W; 790–840 m a.s.l.; annual average temperature is 13° degree Celsius (range 4–24, January and August, respectively) and annual average precipitation is 371.8 mm (range 5–725, July-December, respectively). It covers a surface area of 1,415 ha of predominantly species-rich Mediterranean landscapes (wood pastures or *dehesa*) that are characterised by a habitat mosaic consisting of cereal crops, fallow, natural pastures (*Agrostis castellana*, *Poa bulbosa*, *Stipa tenacissima*, *Brachypodium distachyon*, *Trifolium glomeratum*, *Bellis annua*, *Bellis perennis*) and scrubland (*Cistus ladanifer*, *Quercus coccifera*, *Thymus vulgaris*, *Rosmarinus officinalis*, *Lavandula latifolia*, *Salvia officinalis*, *Santolina chamaecyparissus*, *Helianthemum syriacum*) with scattered holm oaks (*Quercus ilex*). In all, 75% of the estate is covered by herbaceous vegetation and the other 25% by shrubland. The main quarry species are rabbit (*Oryctolagus cuniculus*), red-legged partridge (*Alectoris rufa*), woodpigeon (*Columba palumbus*) and hare (*Lepus granatensis*). Controls of predators annually remove on average 40 red foxes (*Vulpes vulpes*), 70 feral cats (*Felix domesticus*) and 300 magpies (*Pica pica*). This property also holds more than 150 important and protected bird species including Spanish imperial (*Aquila adalberti*) and golden (*Aquila chrysaetos*) eagles, cinereous vulture (*Aegypius monachus*), pin-tailed (*Pterocles alchata*) and black-bellied (*Pterocles orientalis*) sandgrouse, among others.

### Data collection

We analysed red-legged partridge hunting bags from the period 1998–2011. In the field, we examined all bagged birds, certified and classified them as wild partridges. The annual average hunting yield was 0.77 partridges/ha, 33% of the autumn population (2.31 partridges/ha). From spring (1.14 partridges/ha) to autumn, the population increased 51% (Supporting Information, S4 Appendix). Hunting methods (drives), as well as the ability of hunters and beaters, remained constant over the years. The team at Las Ensanchas worked for profit as all shot partridges were sold as a high-quality food product. A total of three to six people processed the warm, recently bagged birds. Age was determined by an examination of primary feathers and sex by spur characteristics [35–39]. Clear reference patterns from a previously identified bird for each age and sex class (young or adult; female or male) were always available. A wing was taken from all birds for precise measurement in the laboratory to confirm the age and sex determination carried out in the field and thus to verify the class they had been assigned to [37,38].

### Statistical analysis

We used *age ratio* (young/adults) to describe the population age structure, *sex ratio* (male/female) to describe the population sex structure, *adult sex ratio* (adult male/adult female) to describe the adult sex structure, and *juvenile sex ratio* (young male/young female) to describe the juvenile sex structure. We examined scatterplots to gain a thorough understanding of the constraining assumptions imposed by our data set [40]. The lack of data independence stemmed from a homogeneous biological structure, as within the population there is a strong family relationship between individuals. Many partridges in the sample came from the same family or the same lineage. These specimens could be more similar to each other than to individuals from other locations or years that are separated by larger distances or longer time periods.

We associated autumn ratios with the summer age ratios derived from counts conducted in July [15]. The two indexes are very different because of different collection methods. We tested for the biological significance of the results and initially applied different procedures and statistical tests to confirm results and to make robust interpretations. The most robust tests were maintained to simplify the results obtained.

To test ratio independence, we used simple regression analyses. For the response, we used ratios resulting from the 1) integer fraction, 2) proportion = dividend / dividend + divisor, and 3) logarithm fraction. We employed frequency = count individuals of one type; absolute frequency = total sample number individuals; relative frequency = proportion; and class (groups of birds according to age and sex) [41]. We used the Chi-square test to determine ratios that differed from a 1:1 ratio as a reference, and contingency tables to test the null hypotheses that sexes and/or ages were distributed randomly over the years.

We employed simple and multiple regression analyses for the response variables (ratios, proportions, class) explained by other ratios, proportions, class, year, and other independent variables such as density or weather. In addition, to fit age and sex ratio responses, we used (a) logistic regression, with model terms: absolute frequency, sample size, year, density and complementary age or sex; (b) generalized regression models, allowing small data sets and multicollinearity, with different distributions to find the best fit; (c) generalized linear models (GLMs), with different numbers of parameters; and (d) mixed models, which take into account complex covariance structures[42]. All tests were considered significant when P < 0.05 or when similar comparisons were significant and supported tests with P < 0.1.

We applied principles of parsimony for rationalising the model and only variables that contributed significantly were retained. The model did not contain any redundant parameters or factor levels and the number of parameters and data were balanced [41]. The AICc (corrected Akaike information criterion), delta AICc and the Akaike weights were used to assess different models, as well the significance level of models and effects [43]. In the GLMs, we applied the deviance as a measure of the goodness of fit. To build the models, we searched for all factors, covariates and interaction terms that might influence ecological explanations. We then carried out a series of step-wise deletions of any non-significant explanatory variables, factors and interaction terms, and continued to remove terms until the best model was obtained [40,43].

To evaluate the effects of weather on age and sex ratios we used data from Las Terceras, a meteorological station run by the AEMET (Spanish Meteorological Agency, http://www.aemet.es) located near the edge of the estate (300 m). We selected the best rain and temperature data according to the relationship between vegetation growth and partridge parameters, and selected the months and variables that showed the greatest correlation with the partridge population [43].

## Results

**Fig 1:**
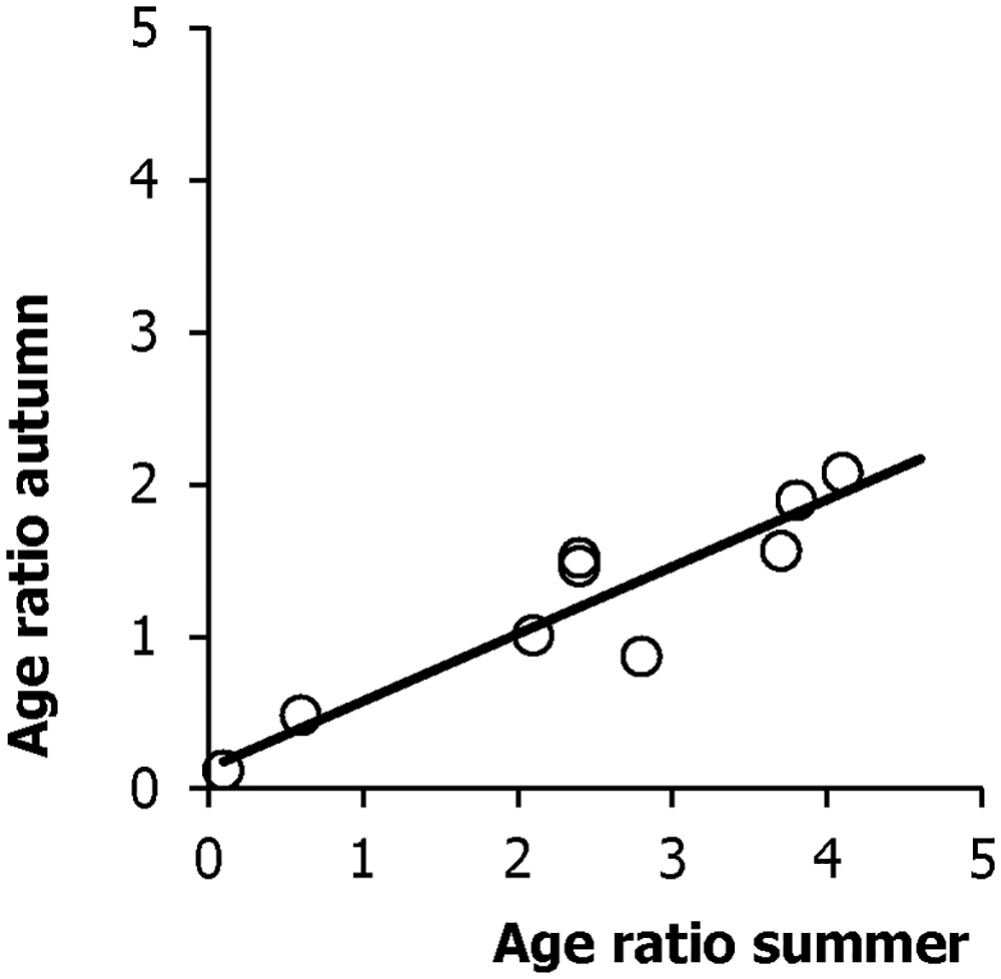
Autumn and summer age ratios in the studied red-legged partridge population.

**Fig 2:**
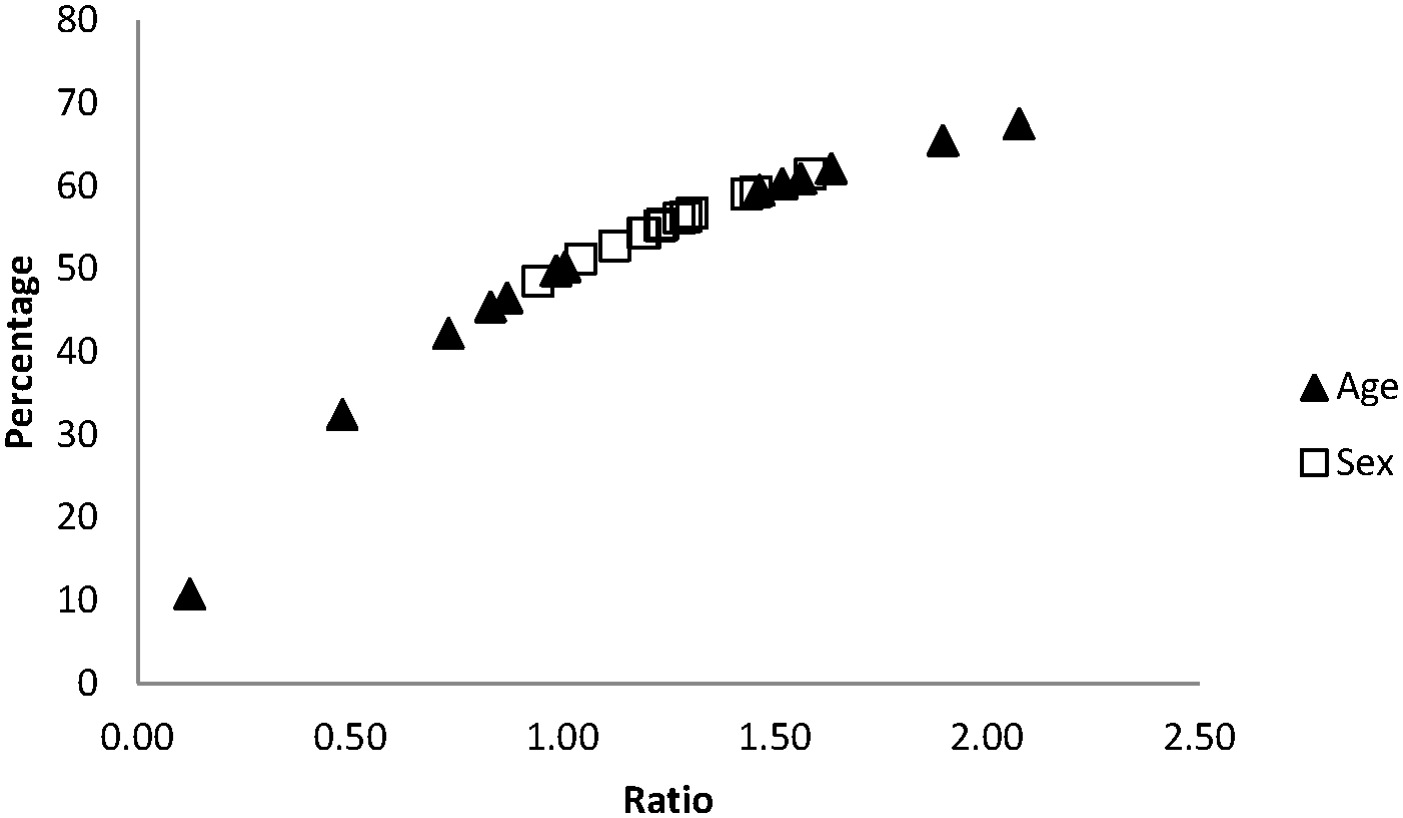
Percentage of juveniles and males in the age and sex ratios in the studied red-legged partridge population.

Autumn age ratios (determined from hunting bags, N = 13813) correlated closely with the summer age ratios (R^2^ = 0.89; P<0.00001) derived from the July car counts (Fig 1). Sex ratio values by their lower variability, were more restricted than age ratios (Fig 2). These ratios were independent of values from previous years. The abundance (partridge/ha yield) was dependent on the sex ratio (R^2^ = 0.31, P<0.04) and percentages of males (R^2^ = 0.31, P<0.04). Density was marginally correlated to age ratios (R^2^ = 0.25, P<0.07) but was better explained by juvenile percentages (R^2^ = 0.35, P<0.02). Age ratios were dependent on sex ratios (R^2^ = 0.31, P<0.04), as well as on the percentages of males (R^2^ = 0.34, P<0.04). We did not obtain similar results with either juvenile or adult sex ratios (Supporting Information, S1 Appendix).

**Fig 3:**
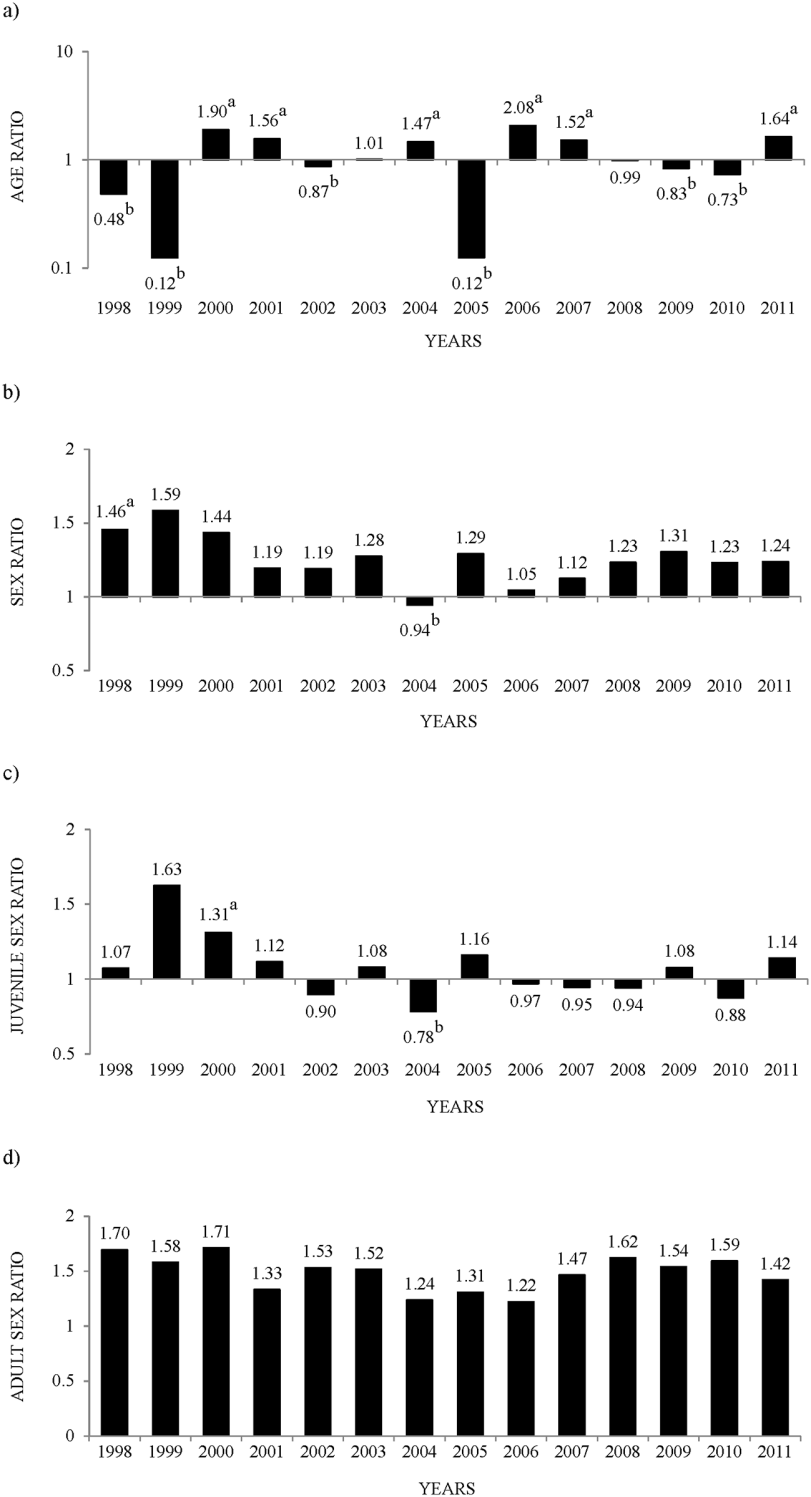
Annual ratios in the studied red-legged partridge population. Superscripts denote a) greater than period mean, b) lower than period mean.

Age ratios changed annually (χ^2^_13_ = 1178.1, P<0.0001) and were higher than the period mean (1.07) in six years, lower in six years and equal the two remaining years (Fig 3a). Similar annual results were obtained when comparing with the 1:1 ratio. Sex ratios displayed some annual changes (χ^2^_13_ = 51.7, P<0.0001), being greater in 1998 and lower in 2004 than the period mean (1.21) (Fig 3b) but similar to this average in the 12 other years. In four years the sex ratio was 1:1 but was greater in the other 10 years. Juvenile sex ratios also exhibited annual changes (χ^2^_13_ = 33.1, P<0.002, N = 7126) and were greater in 2000 and lower in 2004 than the mean (1.01, Fig 3c), and were similar to the average in 12 years. Compared with the 1:1 ratio, juvenile sex ratios were lower in one year, the same in two years and greater in 11 years. Adult sex ratios showed no annual change (χ^2^_13_ = 17.6, P>0.2, N = 6687) and in all years were equal to the mean for the period (1.47) and greater than the 1:1 ratio (Fig 3d).

The model explaining sex was the only one that included a density effect (Table 1, Supporting Information S1, S2 and S3 Appendices). The mixed model used frequency as a response and fixed (age, sex, year, age x sex, age x year, sex x year) and random (age, sex, year x density) effects, generated an AICc = 366.4 with the following significant effects: age (P<0.01), sex (P<0.0004), age x sex (P<0.0002) and age x year (P<0.0005). In multiple regression models, meteorological variables explained 65% of the age-ratio variability and 71% of the adult sex ratio (Tables 2 and 3). Spring cumulative precipitation was positively associated with age ratio and spring temperature with adult sex ratio.

**Table 1:** Generalized linear models (GLMs) for explaining class, sex and age with effects, the corrected Akaike information criterion (AICc) and deviance.

	**Effects**	**AICc**	**Deviance**
**Class**	Absolute frequency**, sample size*	58.17	51.7
**Sex**	Sample size***, age***, year*, density***	17231	17219***
**Age**	Sample size***, sex***, year***	18602	18593***
**Model**	K	Δ_ci_	w_ci_
**Class**	2	0	1
**Age**	3	18543.8	0
**Sex**	4	17172.8	0

Effects: absolute frequency, sample size, age, sex, year, density, significance levels

*0.05,

**0.001 and

***0.0001.

K: number of parameters, Δ_ci:_ corrected Akaike information criterion (AICc) difference between models, w_ci:_ corrected Akaike weights

**Table 2:** Multiple regressions for explaining age ratios and adult sex ratios in terms of meteorological parameters in the studied red-legged partridge population.

	R^2^	F	P	Rain coefficient ±SD	Temperature coefficient±SD	AICc
**Age ratio**	0.65	9.2	0.005	0.006±0.003	0.085±0.047 (minimum)	23.44
**Adult sex ratio**	0.71	12.2	0.002	-0.002*±0.0008	-0.06*±0.018 (average)	-14.44

**Table 3:** Simple regression models for explaining age ratios, juvenile sex ratios and adult sex ratios in terms of meteorological parameters.

Y—X	R^2^	F	P	b±SD	AICc
**Age ratio—January to April rain**	0.53	12.4	0.005	0.019±0.003	22.8
**Age ratio—February minimum temperature**	0.48	11.2	0.006	0.14±0.04	24.4
**Juvenile sex ratio—January to April rain**	0.31	4.9	0.05	-0.002±0.001	-0.8
**Adult sex ratio—February to March rain**	0.36	6.1	0.03	-0.003±0.001	-8.4
**Adult sex ratio—April to June maximum temperature**	0.38	6.1	0.03	-0.05±0.02	-11.0
**Adult sex ratio—April to June average temperature**	0.43	9.0	0.01	-0.07±0.02	-12.1
**Adult sex ratio—April to June minimum temperature**	0.47	10.7	0.007	-0.09±0.03	-13.3

**Fig 4:**
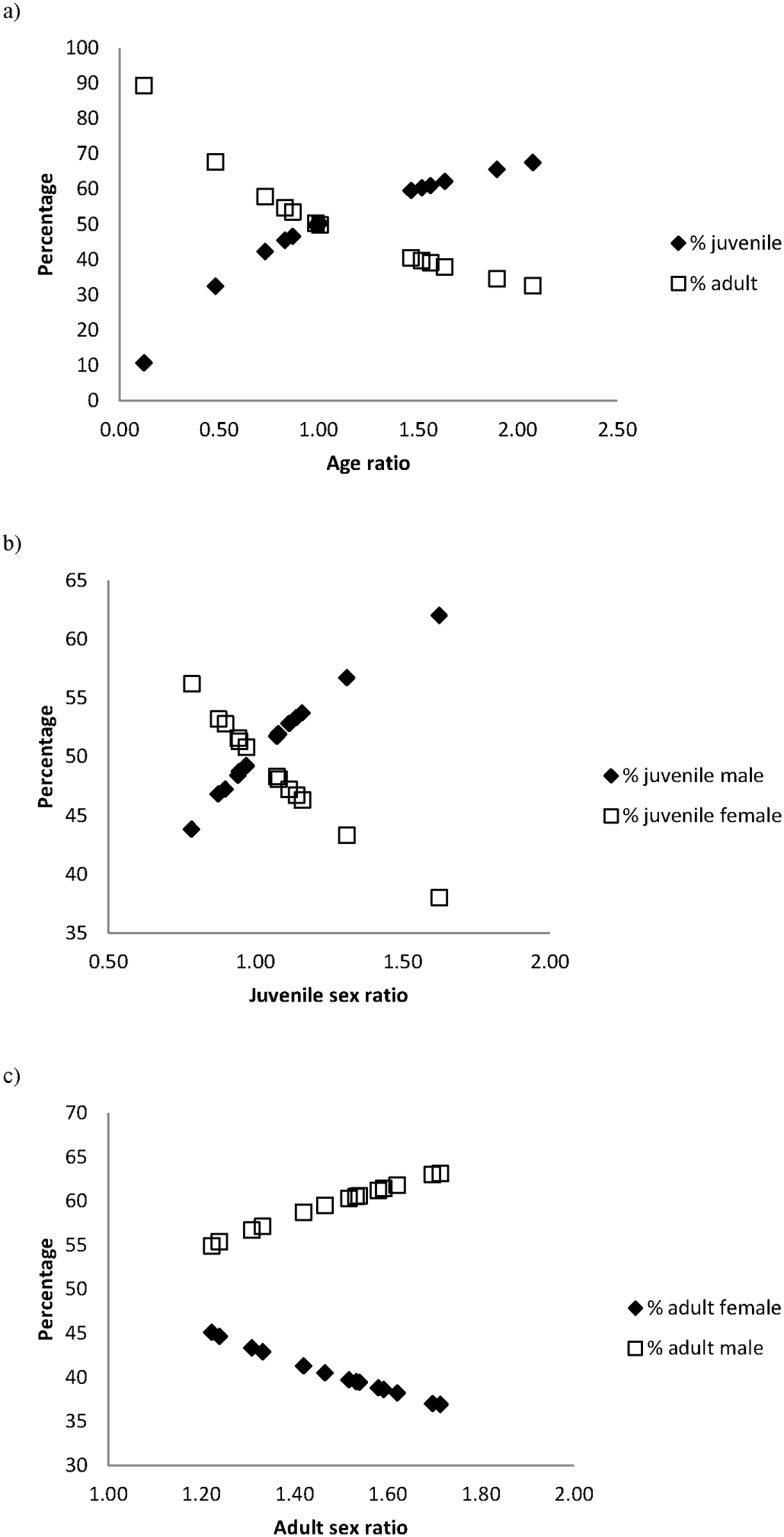
Age, juvenile and adult sex ratios as a percentage of the studied red-legged partridge population.

Age ratio values were broader than sex ratios (Fig 1). Age ratios ranged from 0.12 to 2.08, percentages of young birds from 10.7 to 67.5 and of adults from 32.5 to 89.3 (Figs 3a and 4a). Juvenile sex ratios ranged from 0.78 to 1.63, young male percentages from 43.8 to 62.0, and young female percentages from 38.0 to 56.2 (Figs 3d and 4c). Adult sex ratios ranged from 1.22 to 1.71, adult male percentages from 54.9 to 63.1, and adult female percentages from 36.9 to 45.1 (Figs 3c and 4b).

## Discussion

Sex ratios, chick survival (the key factor in population size), excess males and high chick mortality are all related to population density [2,44,45]. Our findings suggest that there was no significant relationship in ratios between consecutive years [17,22,46]. Density was explained by age (31% of variance) and sex ratios (32% variance) [45], although other factors did have some effect [33,47,48]. Age ratios were dependent on sex ratios (32% of variance) [19,49] and reflected the results of annual productivity minus mortality in the data-collection period. High annual brood production and age ratio variation may mask density-dependent responses [34,50,51]. Abundance partially depends on age and sex ratios [52,53] and in high density population, partridge interactions are more likely and influential. Density depends partially on population structure and age ratios partially depend on sex ratios. The population structure is partially intra-dependent. Inter- and intra-dependence of population structure are partial because these associations can be mediated by other factors (e.g., predators, availability of food and cover, diseases and weather) [54–58].

Predation was responsible for a decrease in the age ratio by half between July and October [1,15,51,55]. Our results showed that 12% more females hatched and had 7.3%-higher juvenile mortality than males, a sexually differentiated mortality that continues into adult age classes. There were 19.2% fewer adult females than adult males, and so the adult sex ratio was male-biased [46,55,59]. The characteristics of the population structure begin to form in the growth stages of partridges (from hatching to juvenile), although the process continues in the maturation phase to adult (from juvenile to adult), with predation being the principal driver of population structure [55,58,60].

From a methodological point of view, all the statistical tools applied were useful for understanding age and sex ratios and the temporal changes they undergo, as well as their relationships with other factors (effects) that contribute to explaining variation. The different statistical tools generate complementary approximations for explaining age and sex ratios. The most complete models explain many different effects, while the simplest show the degree of association [61,62]. For example, our results indicated that multiple regression demonstrates that meteorological parameters explain age and sex ratios, while simple regression illustrates how each meteorological parameter explains these ratios. Age and sex ratio are associated with spring rainfall and temperature. Accordingly, statistical techniques condition our biological interpretation of results and so we must consider how they influence our understanding of wildlife populations to make wide-ranging interpretations. For example, logistic regression for explaining age ratio supports the idea that year and sex had a significant effect, as did the sample size for sex, but density was not included in the effects. Generalized regression models explained the relative frequency with density, year and class effects, while generalized linear models using binomial distribution explained class, sex and age, with effects for absolute frequency, sample size, year and density (Supporting Information S2 and S3 Appendices).

When we manage high-density partridge populations average production and predation maintain close to a 1:1 age ratio in the bag (October). We anticipate that, under future scenarios of adverse meteorological conditions, the NPP will be poorer and will entail a fall in density and age ratios. By contrast, when weather conditions are more favourable, the NPP, density and age ratios will all rise. Partridge populations at high densities maintained a balanced age ratio of around 1:1. Age ratios differed from the period mean (1.07) in 12 of the 14 studied years, thereby indicating that variability in years with lower or higher productivity rates is very frequent. The annual extraction rate should be adjusted to the age ratio of that year [15,63,64].

Sex ratios showed little change over the years, the only variation being change in juvenile sex ratios in some years [33,34]. Accordingly, it is difficult to identify the direct cause behind this population response in the juvenile sex ratio, and it would appear to be the cumulative result of several effects: sex differential in births, mortality, resource availability, predation risk, local storms, local heat waves and other possible causes [9,10,59,65]. Sex ratios only differed from the period mean in two years. Although the juvenile sex ratio showed a little annual change, the adult sex ratio was constant over the entire period and corresponds to the maximum partridge density in a managed habitat, where changes in the availability of resources depend on weather conditions [3,30,66]. In spite of efficient predator control by gamekeepers, adult sex ratios indicate a differential loss of females. Adult females are more exposed to predation than males as a consequence of their parental effort during incubation and chick rearing [18,21,67]. Adult sex ratio distortion in birds is significantly more severe in populations of globally threatened species than in non-threatened species [13,20,23], and greater female mortality is the main driver of male-skewed adult sex ratios in birds [68,69]. Thus, predator control and habitat improvement are needed to balance sex ratios.

We can assume that age ratio homeostasis over decades is the response of a population in a stable habitat where density oscillations occur due to variations in NPP. In high-density partridge populations, longevity increases and production is checked due to higher levels of competition and predation [25,28,53,70]. The same values of age and sex ratios have different population effects in low, middle and high density populations. Consequently, monitoring age and sex ratio controlling for density is necessary to manage and conserve wildlife populations. An useful method for understanding population responses to ecosystem management is required and, accordingly, our study shows how age and sex ratio dynamics are key tools for interpreting, managing and conserving wildlife populations.

## Supporting Information

S1 Appendix Determination coefficient (R^2^) and slopes (b±SD) in regression between abundance (Y) and the age and sex ratios (X). Determination coefficient (R^2^) and slopes (b±SD) in regression between age ratio (Y) and sex ratio (X). Determination coefficient (R^2^) and slopes (b±SD) in regression between age and sex ratio and the previous year age and sex ratio. Effects, corrected Akaike information criterion (AICc), with logistic regression models for age or sex explained by absolute frequency, sample size, year, density, sex or age.
(DOCX)

S2 Appendix Generalized regression models with relative frequency explained by class density, year and class. Generalized regression models with absolute frequency explained by number of trials, class density, year (ordinal), age and sex. Generalized regression models with absolute frequency explained by number of trials, age, sex, year and density. Generalized regression models with absolute frequency explained by number of trials, age, sex, class density and year (ordinal).
(DOCX)

S3 Appendix Generalized linear models (GLMs). Relative frequency, age, sex, year and density. Relative frequency, class, year and density. Absolute frequency, trial, age, sex, year and density. Absolute frequency, trial, class, year and density.
(DOCX)

S4 Appendix Population numbers. (DOCX)
