# Supplementary material for: Age and Sex Ratios in a High-Density Wild Red-Legged Partridge Population
Source: PLoS One. 2016 Aug 10;11(8):e0159765. doi: 10.1371/journal.pone.0159765 (PMC4979962; doi:10.1371/journal.pone.0159765)
Supplement: S1 Appendix — Determination coefficient (R2) and slopes (b±SD) in regression between age ratio (Y) and sex ratio (X). Determination coefficient (R2) and slopes (b±SD) in regression between age and sex ratio and the previous year age and sex ratio. Effects, corrected Akaike information criterion (AICc), with logistic regression models for age or sex explained by absolute frequency, sample size, year, density, sex or age. (DOCX) [file pone.0159765.s001.docx]

Supporting information 1

**Determination coefficient (**R^2^**) and slopes (**b±SD**) in regression between age and sex ratio and the previous year age and sex ratio.**

AR: age ratio, SR: sex ratio, JSR: juvenile sex ratio, ASR: adult sex ratio, AR+1: age ratio next year, SR+1: sex ratio next year, JSR+1: juvenile sex ratio next year, ASR+1: adult sex ratio next year, %J juvenile percentage, %M male percentage, %JM juvenile male percentage, %AM adult male percentage, nl: natural logarithm.

| Y | X | R^2^ | b±SD |
| --- | --- | --- | --- |
| **AR+1** | **AR** | 0.04 | -0.20±0.29 |
| **SR+1** | **SR** | 0.13 | 0.34±0.26 |
| **JSR+1** | **JSR** | 0.04 | 0.20±0.30 |
| **ASR+1** | **ASR** | 0.09 | 0.29±0.27 |

| Y | X | R^2^ | b±SD |
| --- | --- | --- | --- |
| **%J+1** | **%J** | 0.04 | -0.19±0.30 |
| **%M+1** | **%M** | 0.08 | 0.27±0.27 |
| **%JM+1** | **%JM** | 0.03 | 0.17±0.30 |
| **%AM+1** | **%AM** | 0.1 | 0.29±0.27 |

| Y | X | R^2^ | b±SD |
| --- | --- | --- | --- |
| **nlAR+1** | **nlAR** | 0.04 | -0.21±0.30 |
| **nlSR+1** | **nlSR** | 0.08 | 0.27±0.27 |
| **nlJSR+1** | **nlJSR** | 0.02 | 0.14±0.30 |
| **nlASR+1** | **nlASR** | 0.1 | 0.29±0.27 |

**Effects, corrected Akaike information criterion (AICc), with logistic regression models for age or sex explained by absolute frequency, sample size, year, density, sex or age.**

Sex: male, female; Age: juvenile, adult; Absolute frequency: number of individuals, Sample size: number of total individuals, Density: partridge bag by hectare, Model: significance of model, significance levels **0.001 and ***0.0001.

| Y | Effects | Model | AICc |
| --- | --- | --- | --- |
| Sex | Absolute frequency, sample size, year (ordinal)**, age***, density | 0.0001 | 9513 |
| Age | Absolute frequency, sample size***, year (ordinal)***, sex***, density | 0.0001 | 9567 |
