# Supplementary material for: Age and Sex Ratios in a High-Density Wild Red-Legged Partridge Population
Source: PLoS One. 2016 Aug 10;11(8):e0159765. doi: 10.1371/journal.pone.0159765 (PMC4979962; doi:10.1371/journal.pone.0159765)
Supplement: S2 Appendix — Generalized regression models with absolute frequency explained by number of trials, class density, year (ordinal), age and sex. Generalized regression models with absolute frequency explained by number of trials, age, sex, year and density. Generalized regression models with absolute frequency explained by number of trials, age, sex, class density and year (ordinal). (DOCX) [file pone.0159765.s002.docx]

Supporting information 2

**Generalized regression models with relative frequency explained by class density, year and class.**

Probability for effects: class density, year and class, in brackets number of parameters discount.

| Distribution | Normal | Quantile | β | γ | Cauchy |
| --- | --- | --- | --- | --- | --- |
| Estimation method | Maximun likelihood | Maximun likelihood | Lasso | Lasso | Lasso |
| Link function | identity | identity | logit | logarithm | identity |
| AICc | -140.2 | 211.3 | -137 | -133 | -230.3 |
| Density | 0.0001 | 0.0001 | 0.0001 | 0.0001 | 0.0001 |
| Year | 0.0001 | 0.0001 | 0.0001 (-1) | 0.0001 (-2) | 0.0001 |
| Class | 0.001 | 0.4 | 0.003 (-1) | 0.02 (-1) | - |

| Distribution | Binomial | Poisson | Binomial zero inflation | Exponential | Normal |
| --- | --- | --- | --- | --- | --- |
| Estimation method | Lasso | Lasso | Lasso | Lasso | Lasso |
| Link function | logit | logarithm | logit | logarithm | identity |
| AICc | 45.7 | 58.5 | 45.7 | -41.8 | -140.2 |
| Density | - | - | - | 0.0001 | 0.0001 |
| Year | - | - | - | - | 0.0001 |
| Class | - | - | - | - | 0.001 |

| Model | K | ∆_ci_ | w_ci_ |
| --- | --- | --- | --- |
| Exponential | 1 | 188.5 | 0.000 |
| Cauchy | 15 | 0 | 1 |
| γ | 16 | 97.3 | 0.000 |
| β | 17 | 93.3 | 0.000 |
| Normal (Maximun likelihood) | 19 | 90.1 | 0.000 |
| Normal (Lasso) | 19 | 90.1 | 0.000 |
| Quantile | 19 | 441.6 | 0.000 |

K: number of parameters, ∆_ci:_ corrected Akaike information criterion (AICc) difference between models, w_ci:_ corrected Akaike weights

**Generalized regression models with absolute frequency explained by number of trials, class density, year** (ordinal)**, age and sex.**

Probability for effects: class density, year (ordinal), age and sex, in brackets number of parameters discount.

| Distribution | Binomial | β binomial | Poisson | Binomial - | γ  zero inflation |
| --- | --- | --- | --- | --- | --- |
| Estimation method | Lasso | Lasso | Lasso | Lasso | Lasso |
| Link function | logit | logit | logarithm | logarithm | logarithm |
| AICc | 801.1 | 616.6 | 744.2 | 604.7 | 575.2 |
| Density | 0.0001 | 0.0001 | 0.0001 | 0.0001 | 0.0001 |
| Year | 0.0001 | 0.0001(-1) | 0.0001 | 0.3(-10) | 0.02(-10) |
| Age | 0.013 | 0.003 | 0.03 | 0.02 | 0.03 |
| Sex | 0.9 | - | 0.8 | - | - |

| Distribution | Binomial zero inflation | β binomial zero inflation | | β binomial zero inflation | | Normal | | | Normal |
| --- | --- | --- | --- | --- | --- | --- | --- | --- | --- |
| Estimation method | Lasso | Lasso | | Maximun likelihood | | Lasso | | | Maximun likelihood |
| Link function | logit | logarithm | | identity | | identity | | | identity |
| AICc | 801 | 616.6 | | 629,3 | | -3136 | | | -3330 |
| Density | 0.0001 | 0.0001 | | 0.0001 | | - | | | - |
| Year | 0.0001 | 0.0001 | | 0.0001 | | - | | | - |
| Age | 0.01 | | 0.003 | | 0.0006 | | - (-3) | - | |
| Sex | 0.8 | | - | | 0.6 | | - | - | |

| Model | K | ∆_ci_ | w_ci_ |
| --- | --- | --- | --- |
| γ zero inflation | 6 | 0 | 0.999 |
| Binomial - | 6 | 29.5 | 0.000 |
| β binomial | 15 | 41.4 | 0.000 |
| β binomial zero inflation | 16 | 41.4 | 0.000 |
| β binomial zero inflation (Maximun likelihood) | 17 | 54.1 | 0.000 |
| Poisson | 17 | 169 | 0.000 |
| Binomial zero inflation | 17 | 225.8 | 0.000 |
| Quantile | 17 | 225.9 | 0.000 |

K: number of parameters, ∆_ci:_ corrected Akaike information criterion (AICc) difference between models, w_ci:_ corrected Akaike weights

**Generalized regression models with absolute frequency explained by number of trials, age, sex, year and density.**

Probability for effects: age, sex, year and density, in brackets number of parameters discount.

| Distribution | Binomial | β binomial  zero inflation | Poisson | Binomial -zero inflation | γ  zero inflation |
| --- | --- | --- | --- | --- | --- |
| Estimation method | elastic net | Lasso adaptaive | elastic net | elastic net | elastic net |
| Link function | logit | logit | logit | logit | logit |
| AICc | 2184.6 | 663.6 | 1798 | 703 | 679 |
| Age | 0.4 | - | 0.3 | - | - |
| Sex | 0.01 | 0.2 | 0.01 | 0.055 | 0.1 |
| 1998 | - | - | 0.05 | - | 0.02 |
| 2000 | - | - | - | - | - |
| 2001 | - | - | 0.001 | 0.001 | 0.006 |
| 2002 | - | - | 0.001 | 0.001 | 0.002 |
| 2003 | - | - | 0.001 | 0.001 | 0.002 |
| 2004 | - | - | 0.001 | 0.005 | 0.02 |
| 2005 | - | - | - | - | *-* |
| 2006 | - | - | 0.001 | 0.008 | - |
| 2007 | - | - | 0.001 | 0.003 | 0.02 |
| 2008 | - | - | 0.001 | 0.001 | 0.009 |
| 2009 | - | - | 0.001 | 0.07 | - |
| 2010 | - | - | 0.001 | 0.003 | 0.001 |

| Model | K | ∆_ci_ | w_ci_ |
| --- | --- | --- | --- |
| β binomial zero inflation | 1 | 0 | 0.999 |
| Binomial - | 2 | 1521 | 0.000 |
| γ zero inflation | 9 | 15.4 | 0.000 |
| Binomial - zero inflation | 10 | 39.4 | 0.000 |
| Poisson | 12 | 1134.4 | 0.000 |

K: number of parameters, ∆_ci:_ corrected Akaike information criterion (AICc) difference between models, w_ci:_ corrected Akaike weights

**Generalized regression models with absolute frequency explained by number of trials, age, sex, class density and year** (ordinal)**.**

Probability for effects: density, age, sex, class density and year (ordinal).

| Distribution | Binomial | β –binomial | Poisson | Binomial- | γ  zero inflation |
| --- | --- | --- | --- | --- | --- |
| Estimation method | Lasso adaptaive | Lasso adaptaive | Lasso adaptaive | Lasso | Lasso |
| Link function | logit | logit | logit | logit | logit |
| AICc | 801 | 616 | 744 | 604.7 | 575 |
| Age | 0.013 | 0.003 | 0.03 | 0.02 | 0.04 |
| Density | 0.001 | 0.0001 | 0.001 | 0.02 | 0.0001 |
| 1998 | 0.0001 | 0.0002 | - | - | - |
| 2000 | 0.02 | 0.04 | - | - | - |
| 2001 | 0.0001 | 0.0001 | - | - | - |
| 2002 | 0.0001 | 0.0001 | - | - | - |
| 2003 | 0.0001 | 0.0001 | - | - | - |
| 2004 | 0.0001 | 0.0001 | - | - | - |
| 2005 | - | - | - | - | - |
| 2006 | 0.0001 | 0.0001 | - | - | - |
| 2007 | 0.0001 | 0.0001 | - | - | - |
| 2008 | 0.0001 | 0.0001 | - | - | - |
| 2009 | 0.0001 | 0.0001 | - | - | - |
| 2010 | 0.009 | 0.004 | 0.001 | 0.001 | 0.003 |

| Model | K | ∆_ci_ | w_ci_ |
| --- | --- | --- | --- |
| γ zero inflation | 3 | 0 | 0.999 |
| Binomial - | 3 | 29.7 | 0.000 |
| Poisson | 3 | 169 | 0.000 |
| β binomial | 13 | 41 | 0.000 |
| Binomial | 13 | 226 | 0.000 |

K: number of parameters, ∆_ci:_ corrected Akaike information criterion (AICc) difference between models, w_ci:_ corrected Akaike weights
