# Supplementary material for: Age and Sex Ratios in a High-Density Wild Red-Legged Partridge Population
Source: PLoS One. 2016 Aug 10;11(8):e0159765. doi: 10.1371/journal.pone.0159765 (PMC4979962; doi:10.1371/journal.pone.0159765)
Supplement: S3 Appendix — Relative frequency, age, sex, year and density. Relative frequency, class, year and density. Absolute frequency, trial, age, sex, year and density. Absolute frequency, trial, class, year and density. (DOCX) [file pone.0159765.s003.docx]

Supporting information 3

**Generalized linear models (**GLMs**)**

Corrected Akaike information criterion AICc and deviance, significance levels *0.05, **0.001 and ***0.0001.

Relative frequency, age, sex, year and density.

| Distribution | Y | Effects | AICc | Deviance |
| --- | --- | --- | --- | --- |
| Normal | Relative freq | Age**, sex, year***, density***, model*** | -144.5 | 0.09 |
| Binomial | Age | Relative frequency, sex | 24.3 | 19.2 |
| Binomial | Age | Relative frequency, sex, year | 27.5 | 19.1 |
| Binomial | Age | Relative frequency, age, year, density | 30.9 | 18.1 |
| Binomial | Sex | Relative frequency, age | 24.2 | 19.2 |
| Binomial | Sex | Relative frequency, age, year | 27.5 | 19.1 |
| Binomial | Sex | Relative frequency, age, year, density | 30.9 | 18.5* |

| Model | K | ∆_ci_ | w_ci_ |
| --- | --- | --- | --- |
| Binomial Sex | 2 | 0 | 0.415 |
| Binomial Age | 2 | 0.1 | 0.395 |
| Binomial Sex | 3 | 3.3 | 0.080 |
| Binomial Age | 3 | 3.3 | 0.080 |
| Binomial Sex | 4 | 6.7 | 0.015 |
| Binomial Age | 4 | 6.7 | 0.015 |
| Normal Relative ferequency | 5 | 120.3 | 0.000 |

K: number of parameters, ∆_ci:_ corrected Akaike information criterion (AICc) difference between models, w_ci:_ corrected Akaike weights

Relative frequency, class, year and density.

| Distribution | Y | Effects | AICc | Deviance |
| --- | --- | --- | --- | --- |
| Normal | Relative freq | Class**, year***, density***, model*** | 140.0 | 0.09 |
| Binomial | Class | Relative frequency, year, density, Relative frequency x year x density*, model* | 64.0 | 52.8 |

| Model | K | ∆_ci_ | w_ci_ |
| --- | --- | --- | --- |
| Normal | 4 | 76 | 0.000 |
| Binomial | 5 | 0 | 1 |

K: number of parameters, ∆_ci:_ corrected Akaike information criterion (AICc) difference between models, w_ci:_ corrected Akaike weights

Absolute frequency, trial, age, sex, year and density.

| Distribution | Y | Effects | AICc | Deviance |
| --- | --- | --- | --- | --- |
| Binomial | Sex | Trial***, age***, year*, density***, constant*, model*** | 17231 | 17219*** |
| Binomial | Sex | Trial***, age***, year***, constant*, model*** | 18903 | 18893*** |
| Binomial | Sex | Trial***, age***, constant***, model*** | 18704 | 18697*** |
| Binomial | Age | Trial*, sex*, year*, density*, constant*, model* | 17432 | 17420*** |
| Binomial | Age | Trial***, sex***, year***, constant*, model*** | 18602 | 18593*** |
| Binomial | Age | Trial***, sex***, constant***, model*** | 18905 | 18897*** |

| Model | K | ∆_ci_ | w_ci_ |
| --- | --- | --- | --- |
| Binomial Sex | 4 | 0 | 0.000 |
| Binomial Age | 4 | 201 | 0.000 |
| Binomial Age | 5 | -102 | 0.000 |
| Binomial Sex | 5 | 199 | 0.000 |
| Binomial Sex | 6 | -1473 | 1 |
| Binomial Age | 6 | -272 | 0.000 |

K: number of parameters, ∆_ci:_ corrected Akaike information criterion (AICc) difference between models, w_ci:_ corrected Akaike weights

Absolute frequency, trial, class, year and density.

| Distribution | Y | Effects | AICc | Deviance |
| --- | --- | --- | --- | --- |
| Binomial | Class | Absolute frequency**, trial*, model** | 58.17 | 51.7 |
| Binomial | Class | Absolute frequency, trial, year, density | 93 | 51.0 |
| Binomial | Class | Absolute frequency, trial*, year, density, model* | 60.4 | 51.6 |

| Model | K | ∆_ci_ | w_ci_ |
| --- | --- | --- | --- |
| Binomial | 3 | 0 | 0.75 |
| Binomial | 4 | 34.8 | 0.000 |
| Binomial | 5 | 2.23 | 0.25 |

K: number of parameters, ∆_ci:_ corrected Akaike information criterion (AICc) difference between models, w_ci:_ corrected Akaike weights
