# Supplementary material for: Age and Sex Ratios in a High-Density Wild Red-Legged Partridge Population
Source: PLoS One. 2016 Aug 10;11(8):e0159765. doi: 10.1371/journal.pone.0159765 (PMC4979962; doi:10.1371/journal.pone.0159765)
Supplement: S4 Appendix — (DOCX) [file pone.0159765.s004.docx]

Supporting information 4

**Population numbers**
